# A case of disseminated and acyclovir-resistant tumoral herpes simplex virus 2 infection in an immunocompromised patient

**DOI:** 10.1016/j.jdcr.2024.07.028

**Published:** 2024-08-29

**Authors:** Taylor J. Prechtel, Ginat W. Mirowski, Brandon Umphress, Brad E. Rumancik, Honglin Xiao, Rachel P. Kowal

**Affiliations:** a Indiana University School of Medicine, Indianapolis, Indiana; b Department of Dermatology, Indiana University School of Medicine, Indianapolis, Indiana; c Indiana University School of Dentistry, Indianapolis, Indiana; d Department of Pathology and Laboratory Medicine, Indiana University School of Medicine, Indianapolis, Indiana

**Keywords:** acyclovir-resistance, HSV-2 infection, HSV resistance, hypertrophic HSV, pseudotumor HSV, pulmonary HSV, tumoral HSV-2, verrucous HSV

## Introduction

Herpes simplex virus 2 (HSV-2) is one of the most prevalent sexually transmitted infections worldwide.^1^ The seroprevalence of HSV-2 is over 50% in HIV-infected individuals living in the developed world.^2^ In HIV-infected patients, herpetic lesions can be extensive and persistent, with frequent reoccurrences.^3^ Rare hypertrophic, ulcerative, verrucous, and pseudotumoral HSV have been reported in patients with various forms of cellular immune deficiency. However, the majority of these hypertrophic HSV cases occur in the anogenital and oral regions of HIV-infected patients and can be mistaken for malignant neoplasms.^4^, ^5^, ^6^, ^7^, ^8^, ^9^

The standard treatment for HSV-2 infections is acyclovir (ACV) and related nucleoside analogs. ACV resistance ranges from 3.5% to 7% in HIV-infected individuals.^10^ Reported cases of hypertrophic, verrucous, and ulcerative HSV-2 have high rates of ACV resistance and respond poorly to ACV.^9^^,^^11^, ^12^, ^13^, ^14^, ^15^ Treatment alternatives include foscarnet, cidofovir, imiquimod, and thalidomide.^13^^,^^16^, ^17^, ^18^

There is a paucity of cases of tumoral HSV-2 herpetic lesions affecting the face, a few presenting within the respiratory tract, and even fewer presenting with multifocal lesions.^16^^,^^18^, ^19^, ^20^, ^21^, ^22^, ^23^ Herein, we present a case of ACV-resistant pseudotumoral HSV involving the penis, lip, and lung successfully treated with intralesional (IL) cidofovir and intravenous (IV) foscarnet.

## Case report

**Fig 1:**
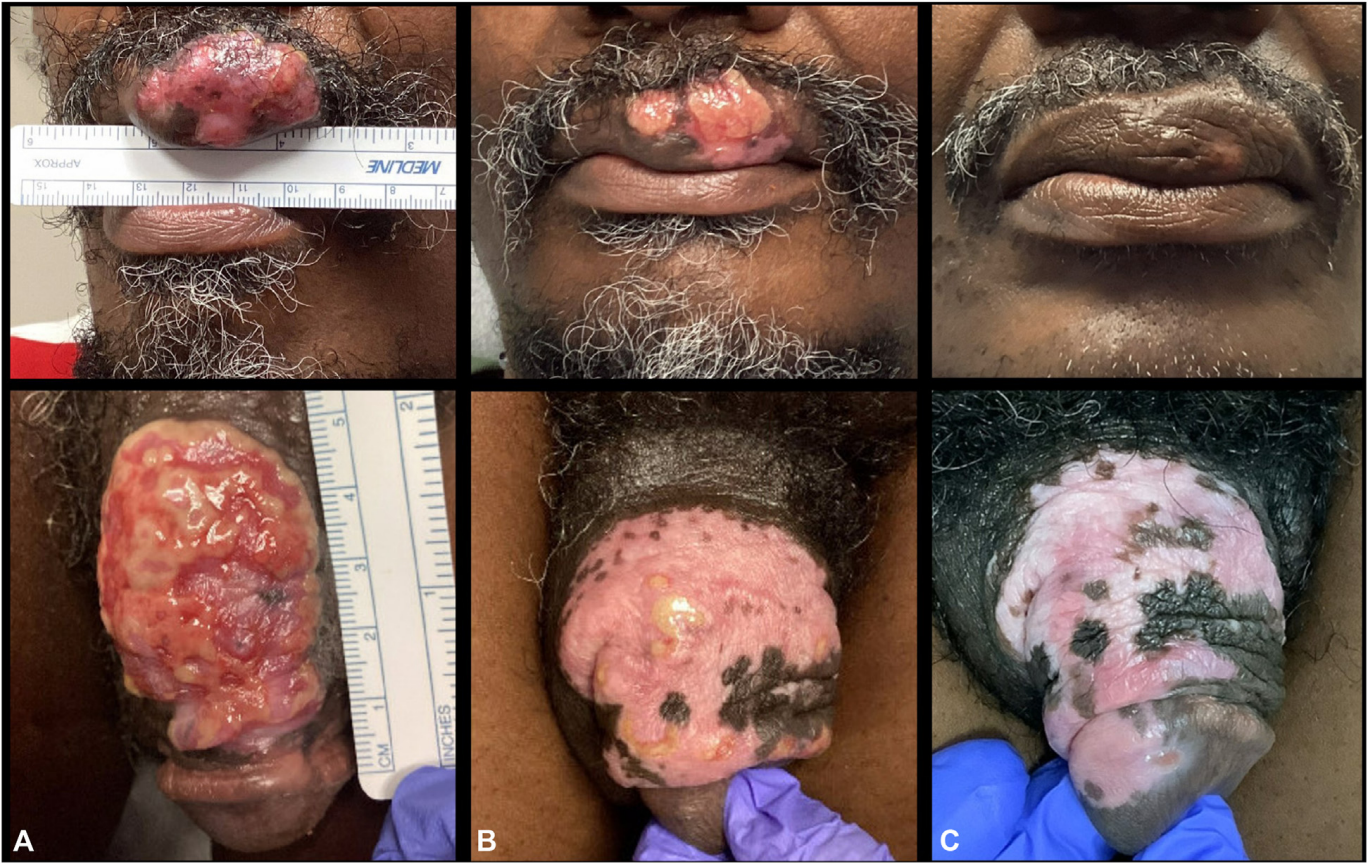
Tumoral HSV-2 infection of left upper vermillion lip and dorsum of the penis. **(A)** Initial presentation, having failed acyclovir, valacyclovir, and a combination of valacyclovir and methylprednisolone. Note lip with 4.5 × 5.2-cm well defined irregular exophytic mass with hypopigmentation, erythema, and focal ulceration, and penis with large 5.0 × 5.0-cm multilobulated, exophytic ulcer with moist wound base. **(B)** After 2 doses of IL cidofovir. Note lip with 3.5 × 2.0-cm pink verrucous plaque with re-epithelialization, re-pigmentation, and thinning of prior noted lesion, and penis with healing verrucous plaque and significant re-epithelization with scattered superficial erosion. **(C)** After 12 days of IV foscarnet and 4 IL cidofovir injections. Note lip with one hypopigmented macule labial aspect of vermillion with no erosions and significant re-epithelialization and re-pigmentation, and penis with extensive hypopigmented macules with incomplete re-epithelization but soft mucosa and no erosions or vesicles.

A 48-year-old man with a past medical history of HIV/AIDS (CD4 count 347 cells/mm^3^, HIV viral load 98 copies/mL at presentation) with intermittent adherence to highly active antiretroviral therapy, herpes labialis, and genital herpes presented with chronic, ulcerative tumor-like HSV on the left upper lip and dorsal shaft of the penis diagnosed by biopsy. Prior therapies included valacyclovir 1 g 3 times daily for 1 month, ACV 400 mg 5 times per day for 1 month, and valacyclovir in combination with methylprednisolone for 1 month. Without improvement, the penile and lip plaques progressed to form multilobulated, exophytic masses (Fig 1, *A*).

**Fig 2:**
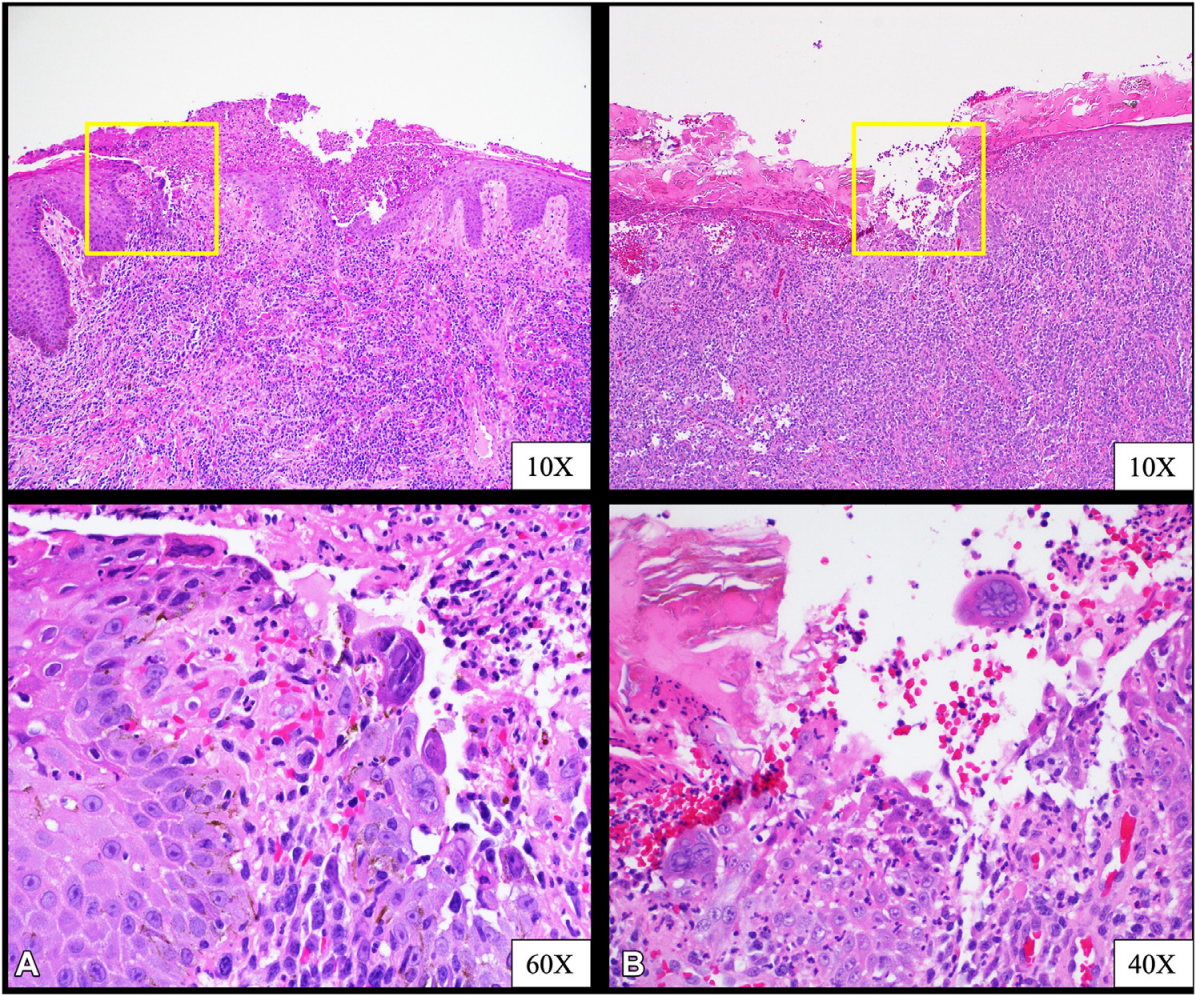
Epidermal ulceration with viral cytopathic effect including multinucleation, margination, and molding, and associated lymphoplasmacytic infiltrate (HE). **(A)** Punch biopsy of dorsal penile shaft: epidermal ulceration with viral cytopathic effect, inflamed. **(B)** Punch biopsy of left upper lip: epidermal ulceration with viral cytopathic effect, inflamed and impetiginized.

Concerns for a neoplastic process or drug-resistant virus were raised in the setting of tumoral presentation and recalcitrance to conventional antiviral treatment. Therefore, repeat punch biopsies were performed, showing viral cytopathic changes and no evidence of neoplasia (Fig 2, *A, B*). Viral tissue culture was positive for HSV-2. Treatment options, including IV or topical cidofovir or inpatient admission for IV foscarnet, were declined by the patient. Instead, he opted for continued valacyclovir therapy for an additional 3 months. With continued tumoral progression, viral sensitivities were obtained and revealed ACV resistance and foscarnet sensitive HSV; cidofovir sensitivities were not reported.

As foscarnet administration required inpatient admission, the patient and clinical team opted for cidofovir 5 mg/kg/dose (450 mg) IV weekly for 3 outpatient sessions. The patient did not demonstrate clinical benefit. Thus, monthly doses of IL cidofovir 1.5% solution were initiated (15 mg/mL; volumes ranged from 1 to 1.5 mL for the upper lip and 3.5 to 4 mL for the dorsal penis). A total of 3 doses resulted in reduction in tumor size, re-epithelialization, and re-pigmentation (Fig 1, *B*). No local or renal side-effects were noted. Despite improvement, but not resolution, the patient was lost to follow-up.

**Fig 3:**
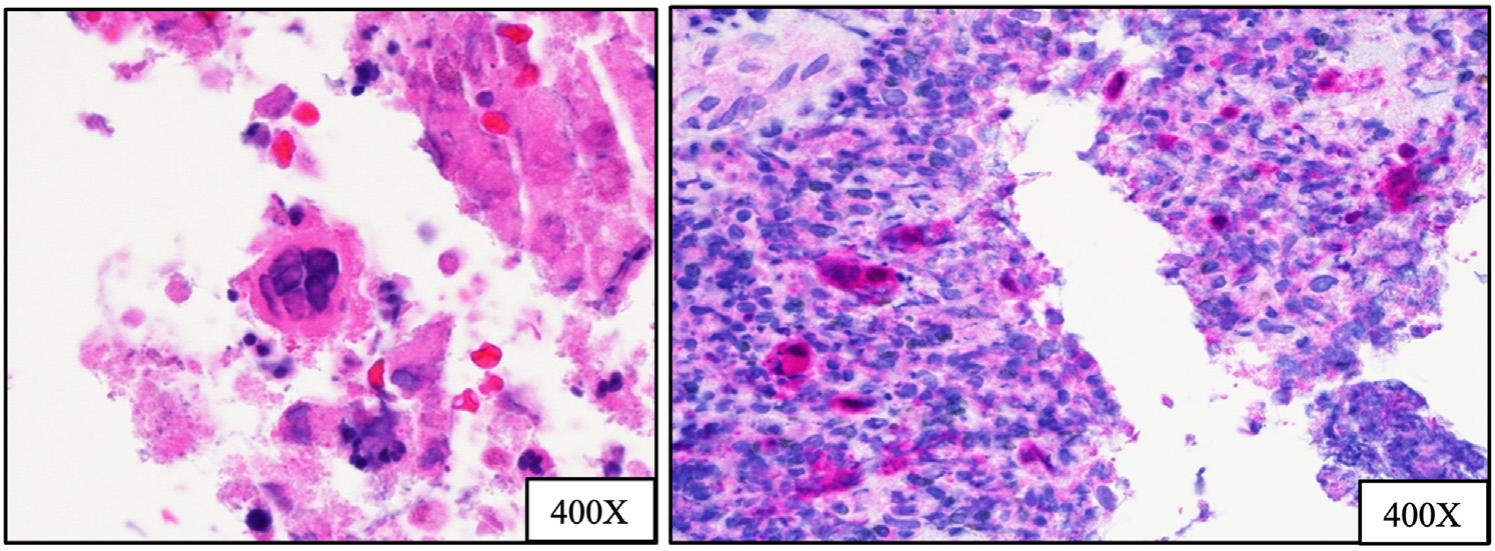
Left mainstem bronchial mass with HSV virally infected cells, and HSV positive immunohistochemical staining (red chromagen).

Seven months later, the patient presented to the emergency department with fever, dyspnea, and stridor requiring urgent intubation and hypoxic respiratory distress, subsequently requiring intensive care unit-level care (CD4 count 119 cells/mm^3^, HIV viral load <20 copies/mL on admission). A subglottic friable mass was found in the posterior larynx with glottic obstruction, along with a left mainstem bronchus mass with post-obstructive pneumonia and prominent mediastinal adenopathy. Tissue obtained from a debulking procedure of the left mainstem bronchial mass revealed extensive necrosis, marked acute and chronic inflammation, no evidence of malignancy, and cells positive for HSV (Fig 3). A 12-day course of IV foscarnet (40 mg/kg Q12) was initiated with a baseline return of the patient’s respiratory status.

At 1-month post-hospital discharge, the lip and penile plaques showed significant re-epithelization. Two superficial 4 mm erosions and rare vesicles were present on the dorsal penis. Thus, the patient received a fourth round of IL cidofovir with almost complete resolution 1 month later (Fig 1, *C*). The patient was scheduled for another injection in 1 month but was unfortunately lost to follow-up.

## Discussion

Herein, we describe a rare case of disseminated, systemic tumoral HSV. Atypical presentations of HSV are well described in immunocompromised hosts, but the multifocal nature of endobronchial and potentially subglottic laryngeal involvement is particularly rare. The first reported case of an HSV-2 endobronchial mass was in 1995 in an AIDS patient successfully treated with ACV.^21^ Upadya et al^22^ describe a case of an HSV-1 endobronchial mass, and Narvaneni et al^23^ report a case of an HSV-1,2 endobronchial pseudotumor. In the latter cases, neither patient had HIV/AIDS and was ultimately transitioned to comfort care before trying antiviral therapy. Roughly 20 cases of laryngeal HSV are present in the literature, with predominant supraglottic involvement, rather than subglottic.^24^ The first case of HSV-1,2 laryngitis in an HIV-infected individual was reported in 1994 and was successfully treated with ACV.^25^ Yang et al^26^ successfully treated an HSV-1 ACV-resistant supraglottic mass with IL cidofovir.

There is a clear, recurrent theme in HSV and HIV-coinfected patients of ACV resistance.^27^^,^^28^ Sensitivity studies should therefore be considered early in refractory plaques to assess for drug resistance, and tissue biopsy is often prudent to rule out malignancy. Pseudotumor lesions are prone to reduced systemic drug delivery.^13^ Intralesional cidofovir has been used successfully for ACV-resistant HSV-related tumors with minimal local or systemic side-effects.^14^^,^^15^^,^^29^ In contrast to ACV, cidofovir and foscarnet are not dependent on the phosphorylation of viral thymidine kinase for activation thus allowing for their use in cases of ACV-resistant HSV.^13^ Additionally, with IL cidofovir, the site of infection receives direct administration of antivirals to achieve a higher local concentration. Finally, the safety profile of IL cidofovir is much improved compared to IV cidofovir due to the avoidance of acute kidney injury, and local side-effects are rarely reported. Our patient experienced dramatic clearance of his skin and lung lesions with IL cidofovir and a 12-day course of IV foscarnet. We attribute our patient’s pseudotumor presentation and eventual dissemination of HSV to the pulmonary system to his immunocompromised state and ACV-resistant HSV.

This case describes ACV-resistant HSV disseminating beyond the lip and penis, causing both pulmonary and potentially laryngeal pseudotumors that responded to IL cidofovir and IV foscarnet. Intralesional cidofovir should be considered for chronic HSV lesions, particularly if the lesions are drug-resistant and recalcitrant to prevent further progression.

## Conflicts of interest

None disclosed.
